# • Impact of relative mental illness on caregivers

**DOI:** 10.1192/j.eurpsy.2021.990

**Published:** 2021-08-13

**Authors:** O. Kazakova, F. Thomas, V. Zrazikova

**Affiliations:** 1 Public Health, Lund University, Malmö, Sweden; 2 Who Collaborating Centre On Culture And Health, University of Exeter, Exeter, United Kingdom; 3 Mathematical And Methodological Support, Institute of Sociology, National Academy of Science, Minsk, Belarus

**Keywords:** Stigma, family care, Belarus, Eastern Europe

## Abstract

**Introduction:**

Belarus is undergoing legislative shifts towards community-based mental health care. Responding effectively to support this process requires an understanding of the experiences and challenges facing families caring for a relative affected by mental illness.

**Objectives:**

To identify how caring for a person with severe mental illness impacts on family carers, and what carers identify as their support needs.

**Methods:**

Semi-structured interviews were undertaken with 17 caregivers of people affected by severe mental illness (diagnosis of F06.8, F20, F25, F7, and/or F 84) in Belarus between March - June 2019.

**Results:**

Care-giving for a family member was usually undertaken on a full time basis with no option for respite. Whilst caring did, in cases, strengthen family solidarity, it also resulted in intensive stress and burnout, financial pressures, and high levels of family tension, exacerbated when the person living with mental illness was perceived as a potential safety risk. High levels of societal stigma meant that care-givers commonly felt unable to discuss their circumstances, travel in public spaces, or participate in community activities. Stigma also deterred carers from seeking professional support. Priorities for support amongst carers included better information, public awareness raising and sensitization, advocacy to support patient integration into social and economic life, peer support and respite for family carers, and an increase in mental health specialists.

**Conclusions:**

Caregiving affected family carers on multiple levels with predominantly negative consequences. Priorities identified by carers need to be considered and acted upon if community-based care is to become an effective option.

